# Two decades of little change: An analysis of U.S. medical school basic science faculty by sex, race/ethnicity, and academic rank

**DOI:** 10.1371/journal.pone.0235190

**Published:** 2020-07-31

**Authors:** Christopher L. Bennett, Raquel Y. Salinas, Joseph J. Locascio, Edward W. Boyer

**Affiliations:** 1 Brigham and Women’s Hospital, Boston, Massachusetts, United States of America; 2 Massachusetts General Hospital, Boston, Massachusetts, United States of America; 3 Harvard Medical School, Boston, Massachusetts, United States of America; 4 The University of Texas MD Anderson Cancer Center UTHealth Graduate School of Biomedical Sciences, Houston, Texas, United States of America; CUNY School of Medicine, City College of New York, UNITED STATES

## Abstract

To examine changes in U.S. medical school basic science faculty over the last 20 years (1998–2018), we undertook an observational study utilizing data from the American Association of Medical Colleges Faculty Roster. Rank (Instructor, Assistant Professor, Associate Professor, and Professor), sex (Female), and race/ethnicity (Asian, Black or African American, Hispanic, Latino, Spanish Origin, or Multiple Race-Hispanic, and White) were analyzed; this reflected a population of 14,047 (1998) to 18,601 (2018) faculty. Summary percent of faculty in various gender, race/ethnicity origin categories were analyzed across years of the study using regression models. We found that females (24.47% to 35.32%) were underrepresented at all timepoints and a minority of faculty identified as Black or African American (1.57% to 1.99%), Hispanic, Latino, Spanish Origin, or Multiple Race-Hispanic (3.03% to 4.44%), or Asian (10.90% to 20.41%). The largest population at all time points was White Male Professors (30.53% to 20.85%), followed by White Male Associate Professors (15.67% to 9.34%), and White Male Assistant Professors (13.22% to 9.75%). Small statistically significant increases were observed among female faculty and faculty at multiple ranks who identified as Black or African American or Hispanic, Latino, Spanish Origin, or Multiple Race-Hispanic. We then completed secondary analyses looking at the interaction of race/ethnicity and Gender. We found: (1) a significant increase (p<0.0001) in both genders who identify as Asian although males had a higher rate of increase (6 point difference, p<0.0001); (2) a significant increase for Black or African American females (P<0.01) not found among males; (3) significant increases (p<0.0001) among both genders of faculty who identify as Hispanic, Latino, Spanish Origin, or Multiple Race-Hispanic although females had an approximately 1% higher rate of increase; and (4) among faculty who identify as White, males had a significant decrease (p<0.0001) while females demonstrated an increase (p<0.0001).

## Introduction

In complex problem solving, diverse teams outperform homogenous teams because the former can capitalize on their unique perspectives and innovative approaches [1–3]. This idea has been extended to the biomedical sciences [2] that scientists from diverse backgrounds and life experiences collectively offer a unique complement of innovation, creativity, and problem-solving abilities that more effectively address complex scientific problems. Despite the added value that diversity brings to scientific endeavors, the science and medicine workforce does not reflect the diversity of our nation’s populace [4–12]. Further, previous work demonstrates declining racial and ethnic representation in clinical academic medical faculty [5]. The U.S. biomedical workforce, including academic healthcare faculty, has traditionally comprised White males and has long underrepresented women and racial and ethnic minorities, such as Black or African Americans, Hispanics or Latinos, American Indians or Alaska Natives, Native Hawaiians and other Pacific Islanders.

A variety of interventions have promoted the entrance of underrepresented minority individuals into the biomedical sciences, such as the National Institutes of Health (NIH) programs Maximizing Access to Research Careers (MARC), Postbaccalaureate Research Education Program (PREP), and F31 Diversity Fellowships, among others. These programs aim to increase the number of underrepresented scientists by facilitating entry into the training pipeline, and while many of these programs have reported varying levels of success [13, 14], they seem to have little effect on the national landscape of diversity at the faculty ranks [15, 16]. This may be due to the fact that few programs exist to specifically target the retention of these individuals at the academic faculty level. These programs tend to focus on supporting transitions into independent faculty roles, such as NIH K01, K99, and diversity supplement programs targeted specifically at underrepresented minority scientists. In addition, despite clear indications of the value that diverse individuals bring to scientific endeavors, considerable evidence suggests that faculty from underrepresented groups face challenges to their entry and retention in academia. For example, when underrepresented individuals finish their PhDs, they are less interested in academic faculty positions at research intensive universities than their well-represented counterparts, even when controlling for career interests at onset of PhD, productivity in scholarship, or research self-efficacy [17]. In addition, women and underrepresented faculty may not feel as welcome or supported in their scientific endeavors [18, 19] and may face systemic barriers (i.e. lack of early career support; funding disparities) that well-represented colleagues do not [20–24].

Given the efforts by policy makers to increase representation of women and traditionally underrepresented racial and ethnic minorities in the U.S. biomedical workforce, we sought to determine trends in faculty demographics and academic rank over time. We examined 20-year (1998–2018) trends in sex, race/ethnicity and faculty rank using data obtained from the American Association of Medical Colleges (AAMC) Faculty Roster. We provide evidence that efforts to diversify faculty composition for woman and traditionally underrepresented racial and ethnic minorities has made little change in the overall faculty demographics, particularly at the professor rank within the AAMC population. Coupled with the fact that the proportion of the U.S. population that is Black or Hispanic has increased [25], the inability of concerted efforts to increase the pipeline of diverse scientists who potentially could enter and move through the academic medical faculty rank, makes these observations particularly alarming. This work highlights the need to augment diversity initiatives that go beyond increasing the number of women and diverse biomedical scientists into the biomedical training pipeline, such as targeted interventions aimed at addressing faculty hiring and start-up package disparities, tenure promotion policies, and faculty retention efforts.

## Methods

### Faculty data

We obtained via online data request twenty years (1998 to 2018) of comprehensive demographic information for U.S medical school faculty collected by the American Association of Medical Colleges (AAMC) Faculty Roster. The data contain voluntarily reported information on full-time faculty at all U.S. medical schools who report to the AAMC; the database includes sex, race/ethnicity, and rank and is presented by Department. The AAMC roster includes both basic and clinical science faculty and is updated annually. The subpopulation of interest was basic science faculty (inclusive of Anatomy, Biochemistry, Microbiology, Pathology-Basic, Pharmacology, Physiology, and Other Basic Science) at the faculty ranks of Instructor, Assistant Professor, Associate Professor, and Professor; we excluded all others (such as Public Health and those from Clinical, Veterinary, and Social Science disciplines). The self-reported demographic characteristics of interest were sex (Male and Female), race/ethnicity(Asian, Black or African American, Hispanic, Latino or of Spanish Origin, Multiple Race-Hispanic, and White). In a similar fashion to previous work, we combined Hispanic, Latino, or of Spanish Origin and those of Multiple Race-Hispanic [4]. Notably, nativity (U. S. born or non-U. S. born) is not reported in this dataset. We calculated proportions for sex, race/ethnicity, and rank of basic science faculty for each year for these groups. Given the extremely low numbers of basic science faculty examined who reported as American Indian or Alaskan Native (less than 0.1% of faculty in 1998 and less than 0.2% of faculty in 2018) and Native Hawaiian or Other Pacific Islander (less than 0.1% of faculty in 1998 and less than 0.1% of faculty in 2018), we did not examine trends in these groups. Overall population changes reflected faculty percentages.

### Statistical analyses

The unavailability of information linking observations across time for the same subject raised concerns that significance tests for common longitudinal methods would be biased and therefore were not employed. Thus, we analyzed the summary percent of faculty in various gender, race, and ethnic categories across years of the study as the dependent variable in analyses. We employed regression methods that estimated and removed possible autocorrelation which would otherwise bias significance tests. We completed analyses looking only at percent versus linear time (years of the study), with others simultaneously examining Time, Academic Rank, and the interaction of: Rank and Time and the interaction of Race/Ethnicity and Gender. In each case, we ran a preliminary test of the significance of four lags of autoregressive autocorrelation with a backward elimination algorithm removing nonsignificant terms, and a Durbin-Watson test of autocorrelation was also applied. We could not include higher order polynomial terms for time beyond the linear, i.e., quadratic and cubic polynomials, in the models because of high collinearity compared to the relatively low degrees of freedom. We examined residuals from fit models graphically to ensure that they were reasonably normally distributed in conformance with significance test assumptions. SAS statistical software (version 9.4) was utilized for analysis; figures were created using the ggplot2 package [26] in R 3.5.1 [27]. The institutional review board at Partner’s Health Care, Boston, Massachusetts, approved the study.

## Results

### Overall population changes

**Fig 1:**
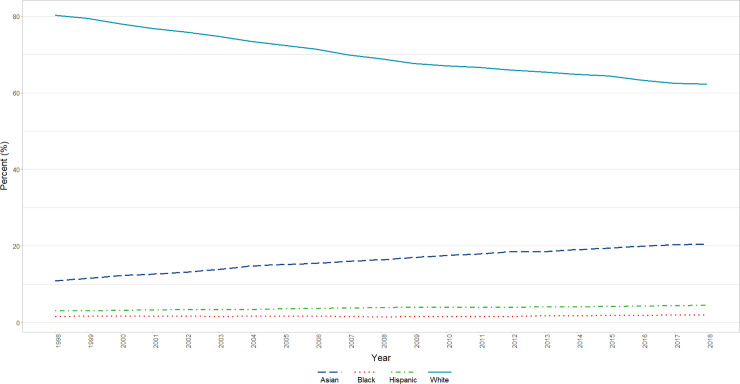
Overall changes in race/ethnicity by year (1998–2018). Graphical representation of raw percentages, unadjusted for autocorrelation, of full-time basic science faculty by race/ethnicity at U.S. Medical schools from December 31, 1998 to December 31, 2018.

The total number of basic science faculty professors, associate professors, assistant professors, and instructors for the period ranged from 14,047 in 1998 to 18,601 in 2018. In 1998, females were underrepresented (24.47%) and a minority of these faculty identified as Black or African American (1.57%), Hispanic, Latino, Spanish Origin, or Multiple Race-Hispanic (3.03%), or Asian (10.90%) in this population. The largest group in this population at that time was White Male Professors (74.62% of all Professors analyzed and 30.53% of the overall population analyzed), followed by White Male Associate Professors (61.91%% of all Associate Professors analyzed and 15.67% of overall population analyzed) and White Male Assistant Professors (46.94% of all Assistant Professors analyzed and 13.22% of overall population analyzed). In 2018, female faculty were still a minority (35.32%) and a minority of faculty identified as Black or African American (1.99%) or Hispanic, Latino, Spanish Origin, or Multiple Race-Hispanic (4.44%). More faculty identified as Asian (20.41%). The largest group in this population in 2018 remained White Male Professors (55.61% of all professors analyzed and 20.85% of the overall population analyzed), followed by White Male Associate Professors (38.34% of all Associate Professors analyzed and 9.34% of overall population analyzed) and White Male Assistant Professors (29.44% of all Assistant Professors analyzed and 9.75% of overall population analyzed). Overall trends (by race/ethnicity) are presented in Fig 1.

### Changes in sex

**Fig 2:**
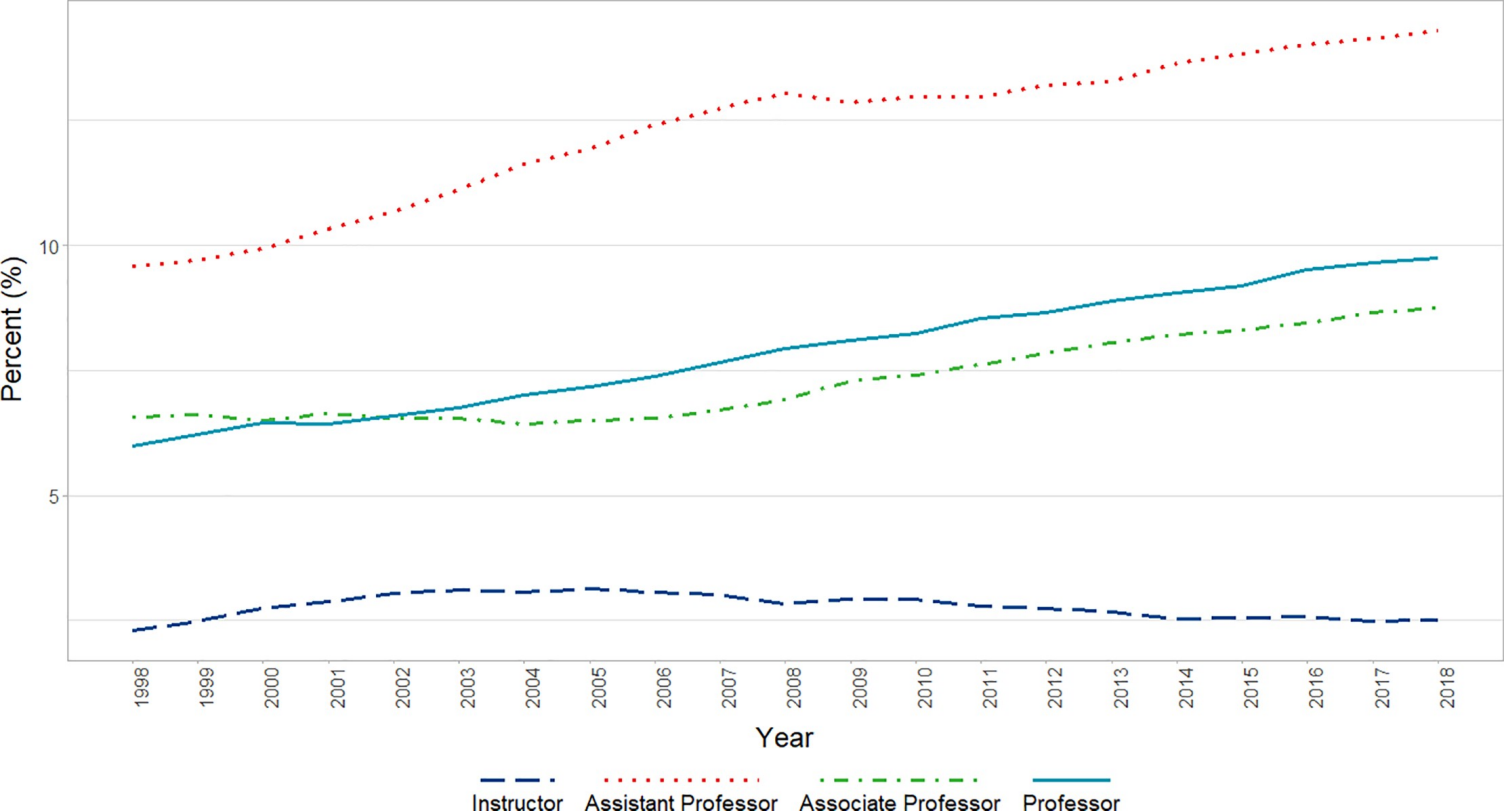
Changes in percent female faculty by year and rank (1998–2018). Graphical representation of raw percentages, unadjusted for autocorrelation, of full-time basic science faculty at U.S. Medical schools from December 31, 1998 to December 31, 2018; reported by faculty who identify as Female at the rank of Instructor, Assistant Professor, Associate Professor, and Professor.

In the population of faculty Instructors, Assistant Professors, Associate Professors, and Professors who identified as Asian, Black or African American, Hispanic, Latino, Spanish Origin, or Multiple Race-Hispanic, or White, a significant (p<0.0001) positive linear effect of time was found where the percentage of faculty who identified as female increased (β = partial unstandardized regression coefficient of percentage versus Year = 0.5428, i.e., the percent was estimated to increase 0.5428 points per year), adjusted for a significant (p = 0.001) positive first order autoregressive (AR1 β = 0.9099) autocorrelation effect. In the parallel analyses that included Academic Rank and its interaction with Time, in addition to a significant (p<0.0001) positive AR1 term (β = 0.9390), a significant interaction of Rank X Time was found (p<0.004), reflecting positive increases across time for all Ranks (Professors, Associate Professors, and Assistant Professors) except for Instructors which had a near zero slope that was significantly different from that of each of the other ranks. (Residuals were reasonably normally distributed for all analyses) (Fig 2).

### Changes in race/ ethnicity

#### Asian

**Fig 3:**
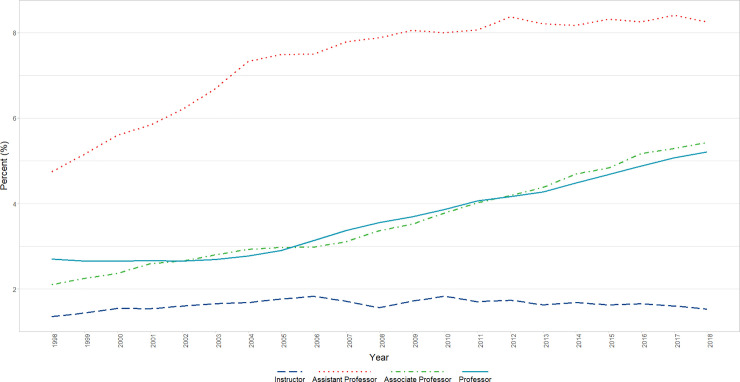
Changes in percent Asian faculty by year and rank (1998–2018). Graphical representation of raw percentages, unadjusted for autocorrelation, of full-time basic science faculty at U.S. Medical schools from December 31, 1998 to December 31, 2018; reported by faculty who identify as Asian at the rank of Instructor, Assistant Professor, Associate Professor, and Professor.

Over the period analyzed, we identified a significant increase in the percentage of faculty who identified as Asian (β = 4779, p<0.0001) after adjusting for AR1 (β = 0.8436, p = 0.0002). In analyses that included Rank, before adjustment for autocorrelation, we found significant increases in the percentage of Asian faculty Professors (P < 0.001), Associate Professors (P < 0.001), and Assistant Professors (P < 0.001), but no significant change among Instructors who identified as Asian (Fig 3). After adjusting for a significant AR1 term (β = 0.95, p<0.0001), a significant (p<0.01) interaction of Rank and Time was found reflecting significant increases for Associate and Assistant Professors but near flat effects for Professors and Instructors (Fig 3).

#### Black or African American

**Fig 4:**
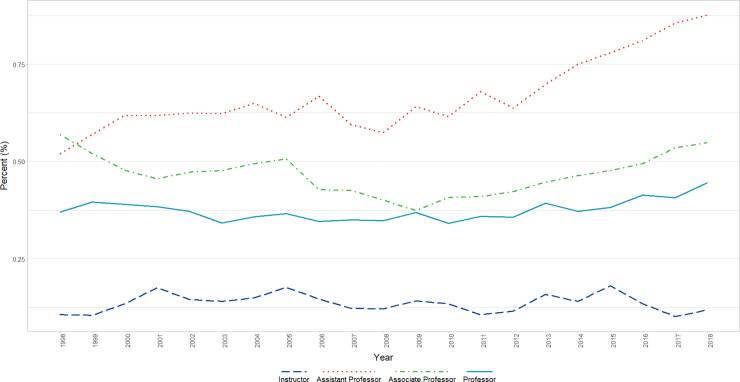
Changes in percent black or African American faculty by year and rank (1998–2018). Graphical representation of raw percentages, unadjusted for autocorrelation, of full-time basic science faculty at U.S. Medical schools from December 31, 1998 to December 31, 2018; reported by faculty who identify as Black or African American at the rank of Instructor, Assistant Professor, Associate Professor, and Professor.

For analyses in which percent Black or African American faculty was the dependent variable, although a significant increase in percent across the span of years studied (P<0.05) was present, the trend deviated from a steady straight-line increase. In the analysis of time alone, the positive linear increase across time became only marginally significant (β = 0.02, p = 0.0884) after adjusting for a significant positive AR1 autocorrelative term (β = 0.8716, p<0.0001; the Durbin-Watson test of lag 1 autocorrelation was significant before adjusting for the AR1, but not after); the autocorrelation term absorbed much of the variance due to the deviation from a straight-line trend. In the analyses that included Rank, after adjusting for a significant AR1 (β = 0.8239, p<0.0001), a significant interaction of Rank and Time was found in which there was a significant increase in the proportion of Assistant Professor faculty who identified as Black or African American (P < 0.001); but other Ranks showed relatively flat trends. We found a significant difference in mean percent change across time in comparing that of the Assistant Professors with that of each of the other ranks (p<0.01) (Fig 4).

#### Hispanic, Latino, Spanish Origin, or Multiple Race-Hispanic

**Fig 5:**
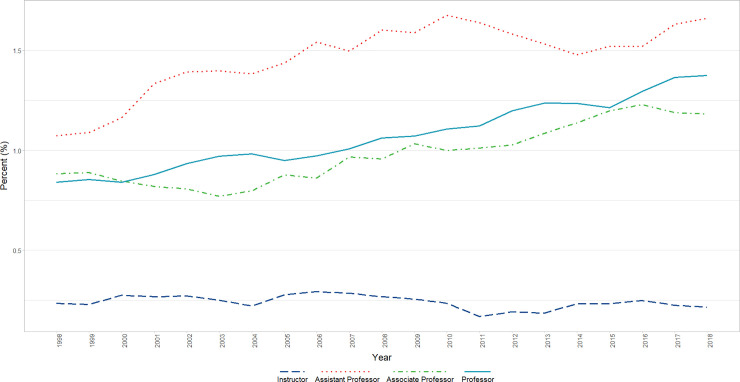
Changes in percent Hispanic, Latino, Spanish Origin, or Multiple-Race Hispanic faculty by year and rank (1998–2018). Graphical representation of raw percentages, unadjusted for autocorrelation, of full-time basic science faculty at U.S. Medical schools from December 31, 1998 to December 31, 2018; reported by faculty who identify as Hispanic, Latino, Spanish Origin, or Multiple Race-Hispanic at the rank of Instructor, Assistant Professor, Associate Professor, and Professor.

For analyses in which percent faculty who identified as Hispanic, Latino, Spanish Origin, or Multiple Race-Hispanic was the dependent variable, a significant increase in percent across the span of years studied (p<0.0001, β = 0.07) remained after adjustment for AR1 (β = 0.62, p = 0.003). In the analyses that included Rank, a significant positive AR1 term and relatively weaker significant negative AR3 term (β = 0.93, p<0.0001, β = −0.23, p = 0.02, respectively), were removed, after which a significant interaction of Rank and Time was found (p<0.001) in which there was a significantly greater increase in the proportion Hispanic, Latino, Spanish Origin, or Multiple Race-Hispanic across time for Associate and Assistant Professors than for Professors and Instructors, both of the latter showing fairly flat trends (Fig 5).

#### White

**Fig 6:**
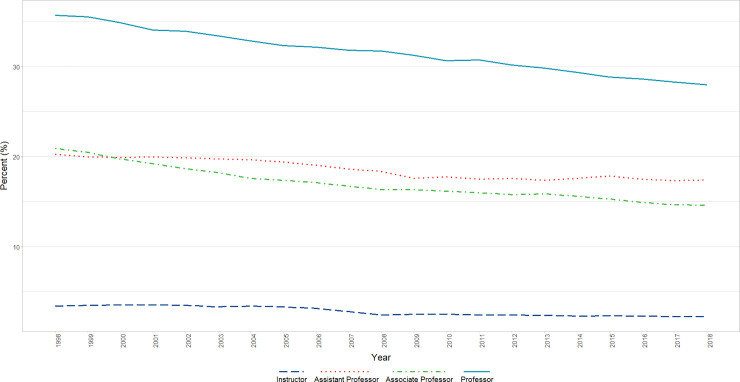
Changes in percent White faculty by year and rank (1998–2018). Graphical representation of raw percentages, unadjusted for autocorrelation, of full-time basic science faculty at U.S. Medical schools from December 31, 1998 to December 31, 2018; reported by faculty who identify as White at the rank of Instructor, Assistant Professor, Associate Professor, and Professor.

We found a significant decrease in the percentage of faculty who identified as White, which was significant (β = −0.9, p<0.0001) after adjusting for AR1 (β = 0.9263, p<0.0001). In analyses that included Rank, a significant positive AR1 term and relatively weaker significant negative AR4 term (β = 0.9747, p<0.0001, β = −0.204, p = 0.0025, respectively) were removed, after which a significant (p<0.05) interaction of Rank and Time was found in which there were significant decreases in the percentage of all ranks: White Professors (P < 0.001), Associate Professors (P < 0.001), and Assistant Professors (P < 0.001), but weakest for Instructors (P < 0.05) (Fig 6).

### Interactions between race/ethnicity and gender

#### Asian

**Fig 7:**
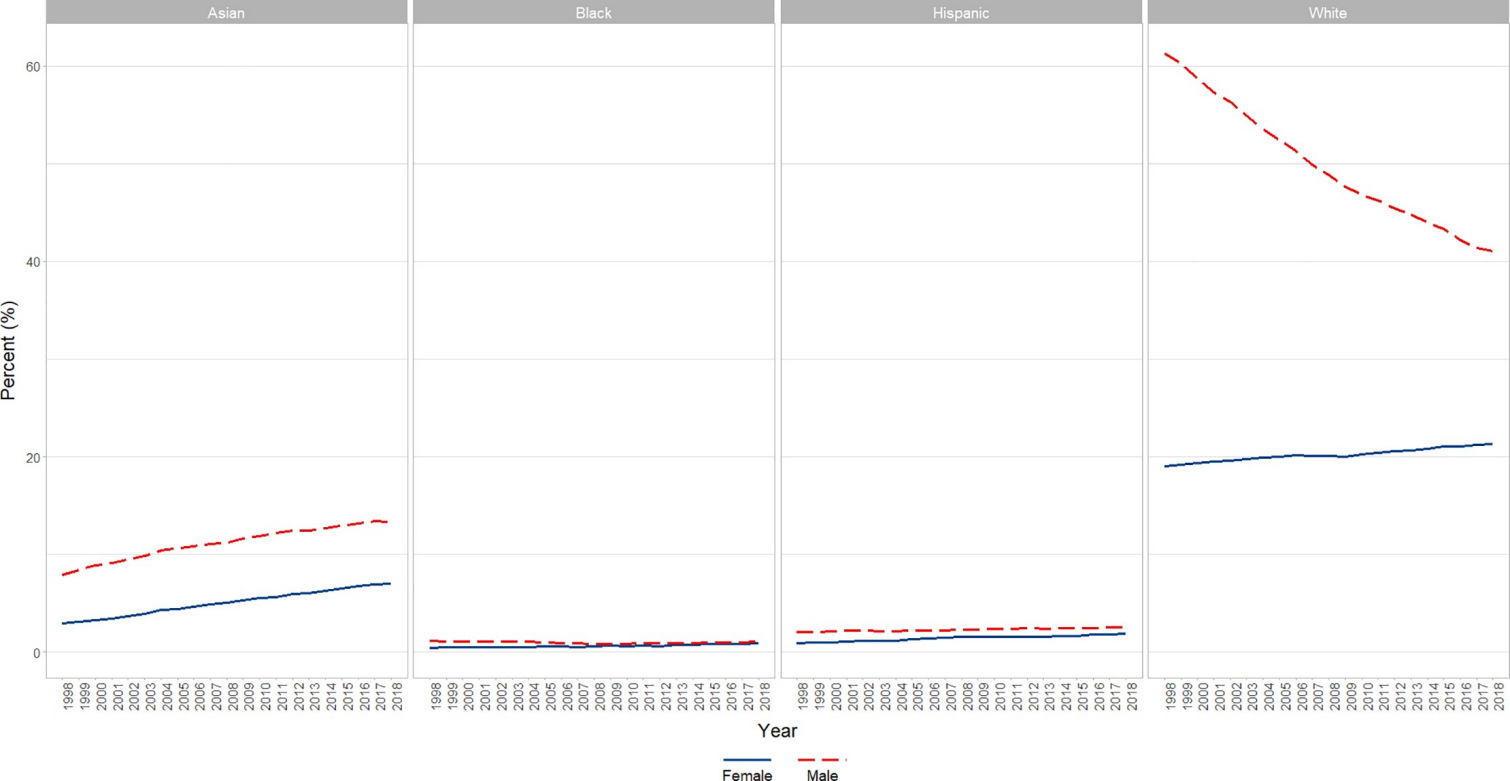
Overall changes in percent female faculty by self-reported race/ethnicity over time (1998–2018). Graphical representation of overall change in raw percentages, unadjusted for autocorrelation, of full-time basic science faculty at U.S. Medical schools from December 31, 1998 to December 31, 2018; separated by race/ethnicity.

**Fig 8:**
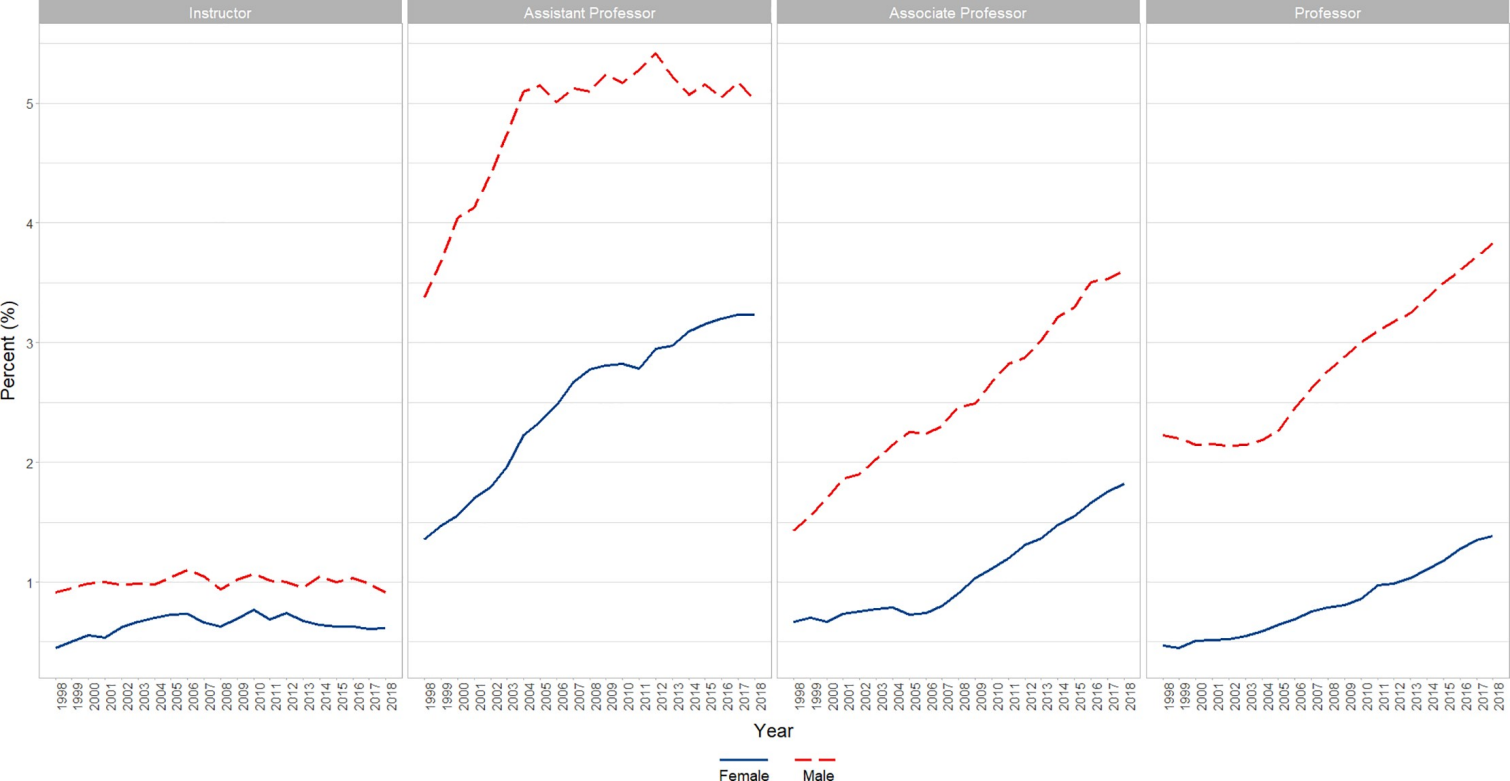
Changes in percent Asian faculty by gender and academic rank over time (1998–2018). Graphical representation of overall change in raw percentages, unadjusted for autocorrelation, of full-time basic science Asian faculty at U.S. Medical schools from December 31, 1998 to December 31, 2018 by rank (Instructor, Assistant Professor, Associate Professor, and Professor).

We identified a significant increase over time in the percentage of both male (β = 0.27, p<0.0001) and female faculty (β = 0.212, p<0.0001) who identified as Asian, though Males had a significantly higher percent overall across time (6 point difference; p<0.0001). Male and Female faculty differed slightly but significantly in slope (Males higher slope; p<0.0001) without autocorrelation (first and second lag) adjustment; a finding that reversed after autocorrelation adjustment (Females higher slope; p<0.0001) (Figs 7 and 8).

#### Black or African American

**Fig 9:**
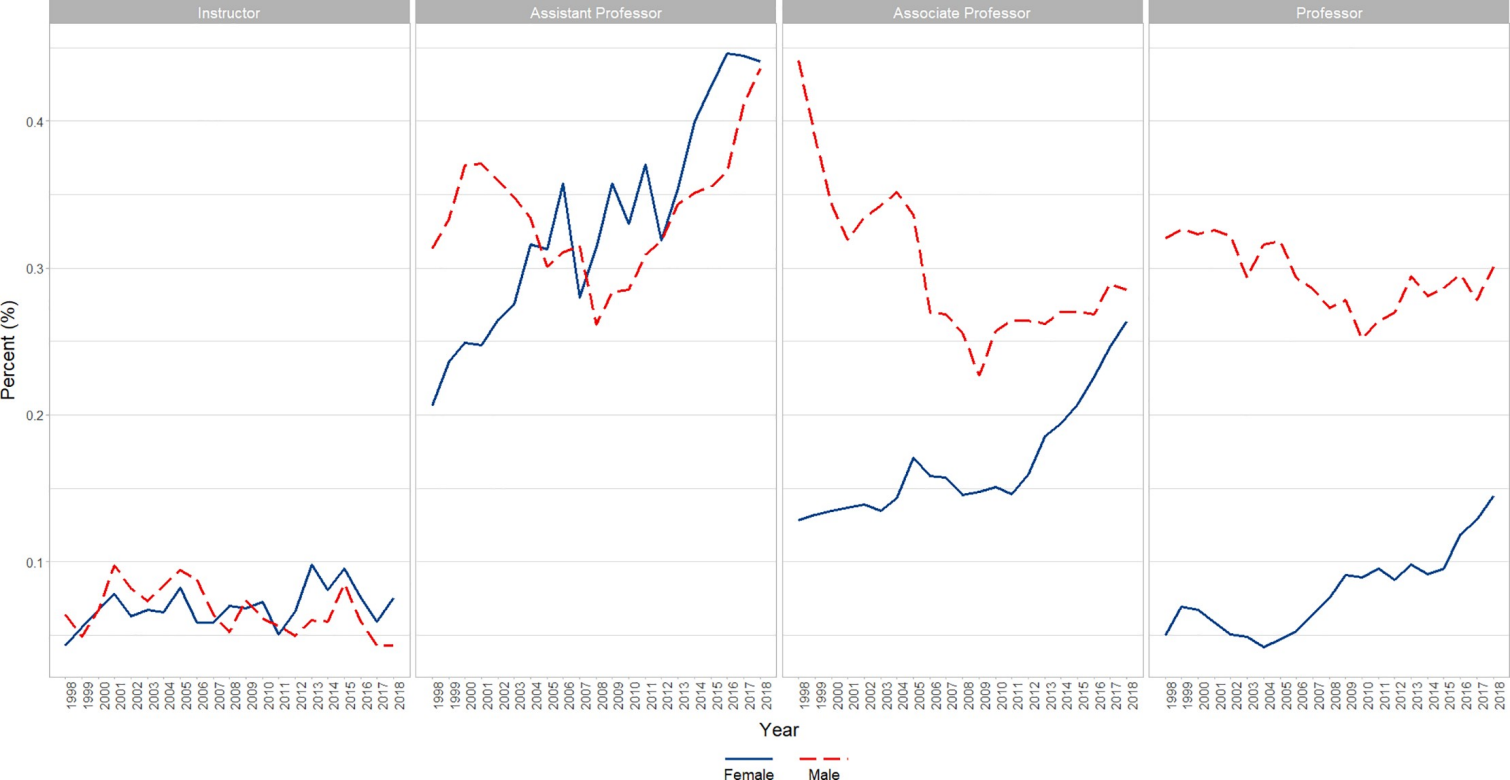
Changes in percent black or African American faculty by gender and academic rank over time (1998–2018). Graphical representation of overall change in raw percentages, unadjusted for autocorrelation, of full-time basic science Black of African American faculty at U.S. Medical schools from December 31, 1998 to December 31, 2018 by rank (Instructor, Assistant Professor, Associate Professor, and Professor).

We ran an analysis of percentage for Blacks or African American versus Year, Gender, and the interaction of gender x year, adjusting for a significant lag 1 autoregressive term. The interaction term was significant (p = 0.0069) reflecting a significantly (P<0.01) increasing trend for Black or African American Females across years (β = 0.02), whereas Black or African American Males were essentially flat (a nonsignificant slight decrease) (Figs 7 and 9).

#### Hispanic, Latino, Spanish Origin, or Multiple Race-Hispanic

**Fig 10:**
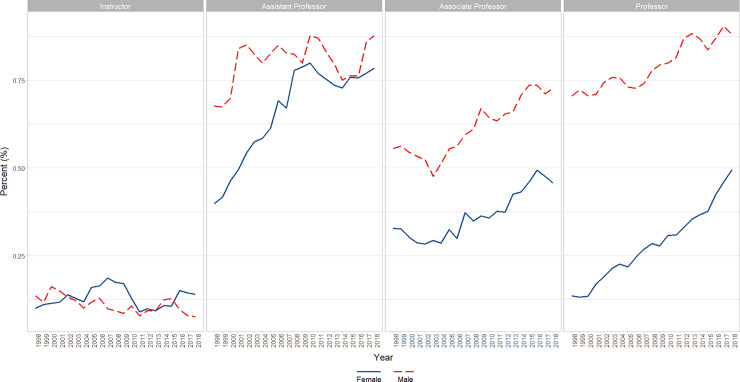
Changes in percent Hispanic, Latino, Spanish Origin, or Multiple Race-Hispanic faculty by gender and academic rank over time (1998–2018). Graphical representation of overall change in raw percentages, unadjusted for autocorrelation, of full-time basic science Hispanic, Latino, Spanish Origin, or Multiple Race-Hispanic faculty at U.S. Medical schools from December 31, 1998 to December 31, 2018 by rank (Instructor, Assistant Professor, Associate Professor, and Professor).

Both genders increase over time, and although females are lower overall (by approx. 1 percentage point; p<0.0001), they increase at a significantly (p<0.0001) higher rate than males over time (0.045 vs 0.02; first and second lag autocorrelation adjusted) (Figs 7 and 10).

#### White

**Fig 11:**
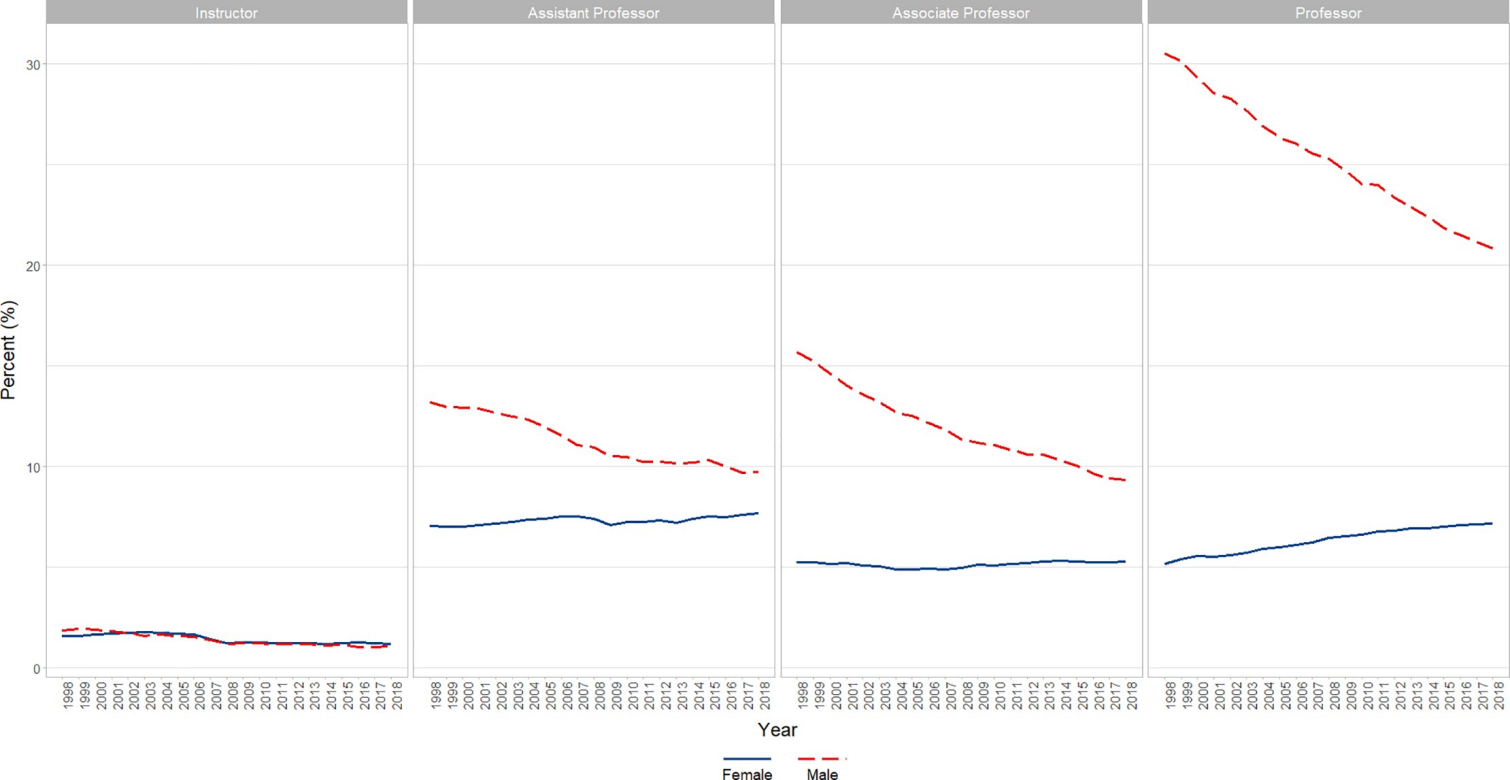
Changes in percent White faculty by gender and academic rank over time (1998–2018). Graphical representation of overall change in raw percentages, unadjusted for autocorrelation, of full-time basic science White faculty at U.S. Medical schools from December 31, 1998 to December 31, 2018 by rank (Instructor, Assistant Professor, Associate Professor, and Professor).

There is a significant (p<0.0001) interaction of gender x year in which males go down significantly (p<0.0001; β = -1.0) to a large degree and the females go up to a small, but significant degree (p<0.0001; β = +0.1). Overall, across time Males are significantly higher than Females (p<0.0001; from about 40 percentage points higher at year 2000 to about 25 at year 2015) (Figs 7 and 11)

## Discussion

We found a significant increase in the number of female basic science faculty at U.S. Medical schools that report to the AAMC faculty Roster both overall and at multiple academic ranks. Males, however, still represent the majority of basic science faculty, overall and at all ranks. These values and the percentages at other academic ranks stand in striking contrast to recent U.S. Census Bureau population estimates that females represent 50.8% of the current U.S. population [25]. Although we also found a significant decrease in proportion of faculty who identify as White (both overall and at all ranks over the twenty years reviewed) and that this paralleled significant increases in the percentage of faculty from traditionally underrepresented groups, it should be taken in the context that: (1) White faculty, overall and at all ranks, were still found to represent the vast majority in all analyses and (2) traditionally underrepresented groups within the faculty population studied are at proportions much lower than those seen among recent U.S. population estimates. Finally, we also examined the interaction of race/ethnicity and gender. Across all faculty rank (except Instructor) and demographic groups, the change in percentage over time for Female faculty modestly increased. Notably, similar trends were observed for Male faculty from traditionally underrepresented groups, except for Black or African American Male faculty. In the case of the latter, no change was observed for those at the Associate or full Professor rank.

Others have noted similar trends in select ranks of basic science faculty. For example, Gibbs et al. [15] examined AAMC faculty roster data and reported that the percentage of underrepresented minority assistant professors in basic science departments in medical schools’ professors grew from 3.9% in 1980 to 5.8% in 2014, and Meyers et al. [28] described no growth for underrepresented individuals in full professors ranks. Our study differs by reporting trends by gender and race/ethnicity across all faculty ranks as well as offering an analysis of the interaction between race/ethnicity and gender. It highlights that gains in representation can be described as modest as best for female and most traditionally underrepresented faculty, except in the case of Black faculty, where no change has occurred over the past 20 years.

Collectively, studies of faculty representation show that gender disparities and underrepresentation is pervasive in basic faculty across the ranks at medical schools. Other studies show similar trends in medical faculty as well [5,6,8–10,12]. In contrast to our current findings, a recent study that: (1) utilized the same dataset and (2) was aimed at evaluating trends in sex race/ethnicity among another population of faculty at U.S. Medical school (clinical specialties) identified worsening underrepresentation in most specialties [5]. This issue of underrepresentation was also found to extend to the precursor of this faculty population (resident physicians) [6, 10]. For basic scientists the rate of PhD attainment for underrepresented individuals has increased significantly over the past 33 years, but the rate at which they enter the assistant professor rank has not [15]. The “decoupling” of these two career stages suggests an intervention point to target in efforts to diversify faculty ranks. Our study further guides thinking in how to address faculty diversity. In addition to targeting the transitions towards the Assistant Professor rank, we also will need to target transitions to later career stages (Associate and full Professor ranks). Collectively these works, and others, demonstrate that issues surrounding diversity are ongoing and not unique to the basic science faculty population [5, 6, 8, 10, 12].

Across the board these studies demonstrate the need to address faculty entrance and retention efforts for female and underrepresented minorities. Some institutions are already experimenting with efforts in this vein. For example, Emory utilized cluster hiring, the process of hiring multiple faculty at one time rather than the usual practice of hiring faculty one by one [29]. The institution was able to hire 81 faculty from 2017–2019 of which 51% were underrepresented minority faculty. Clustering these hires allowed the institution to mitigate a problem faculty of color often report- isolation and/or exclusion [30,31]. Another approach has been to demystify unwritten rules regarding the tenure promotion process through mentorship and targeted professional development [32] It is encouraging to see efforts to support women and faculty of color, but it is presently unclear how widespread the practice is or how it will impact faulty retention. More work to study outcomes will be required.

Several limitations are present in our study. First, we were unable to obtain links indicating which records are from the same subject across time; as such, we assumed independence of observations across time (apart from their connections among variables indicated, i.e., gender, race, etc.) with the understanding that any error produced by this assumption would be conservative error given we were analyzing repeated measures data as if it were independent; this may have reduced power and underestimated significance. This problem is additionally minimized given we analyzed collective percentages at the group level and removed otherwise biasing autocorrelation at that level. Second, AAMC data on gender and race/ethnicity is self-reported. The dataset analyzed did not report nativity and admittedly these factors can influence how faculty navigate their academic environment [30]. Some schools may only report clinical departments, not basic science departments. Further, this data reflects a subpopulation of faculty and may not reflect basic science faculty who work in other departments or those who may: (1) instead be reported elsewhere or in other basic science departments at institutions who do not report to the AAMC or (2) basic science faculty outside the U.S. It is also not inclusive of faculty from Public Health and those from Clinical, Veterinary, and Social Science disciplines which might have completely different findings. These trends, however, likely represent the majority of academic basic science faculty in the U.S. and reflect national level trends of the overall faculty population at research intensive universities. For example, a collective examination of demographic data for all U.S. faculty collected from the Integrated Postsecondary Education Data System (IPEDS) from 2013–2017, showed very modest increases in faculty for women (1.9%) and Black tenured faculty (0.5%), while a slight decrease (0.05%) was observed for Hispanic tenured faculty. Third, although the AAMC reports multiple different departments (such as Biochemistry and Microbiology), given the possibility that many of these departments may overlap, have been fused, renamed, or dissolved over the course of the period study, we chose to focus on the collective basic sciences rather than individualized departments or programs. To our knowledge, we are not aware of any other reporting agency to which basic science departments would otherwise report. This work is, as such, likely a best-fit means to query national level trends in the basic science academic workforce.

## Conclusion

While we found a significant increase in the number of female basic science faculty of all ranks in this AAMC Faculty Roster, basic science faculty at all ranks are largely still comprised by males. Traditionally underrepresented racial/ethnic minorities within the faculty population studied were at proportions much lower than those seen among recent U.S. population estimates and showed the least growth over the time period examined.

As evidenced by these findings, ongoing interventions have done little to increase the numbers of traditionally underrepresented ethnic and racial minorities in this basic science faculty population. Our hope is that continued examination of faculty demographics will prompt universities and policy makers to renew and augment efforts to address systemic barriers to entering and remaining in basic biomedical faculty ranks.
